# Comparative microRNA Transcriptomes in Domestic Goats Reveal Acclimatization to High Altitude

**DOI:** 10.3389/fgene.2020.00809

**Published:** 2020-07-31

**Authors:** Siyuan Feng, Jideng Ma, Keren Long, Jinwei Zhang, Wanling Qiu, Yan Li, Long Jin, Xun Wang, Anan Jiang, Lingyan Liu, Weihang Xiao, Xuewei Li, Qianzi Tang, Mingzhou Li

**Affiliations:** Institute of Animal Genetics and Breeding, College of Animal Science and Technology, Sichuan Agricultural University, Chengdu, China

**Keywords:** miRNA, high-altitude acclimatization, goat, multiple tissue, angiogenesis

## Abstract

High-altitude acclimatization is a representative example of vertebrates’ acclimatization to harsh and extreme environments. Previous studies reported sufficient evidence for a molecular genetic basis of high-altitude acclimatization, and genomic patterns of genetic variation among populations and species have been widely elucidated in recent years. However, understanding of the miRNA role in high-altitude acclimatization have lagged behind, especially in non-model species. To investigate miRNA expression alterations of goats that were induced by high-altitude stress, we performed comparative miRNA transcriptome analysis on six hypoxia-sensitive tissues (heart, kidney, liver, lung, skeletal muscle, and spleen) in two goat populations from distinct altitudes (600 and 3000 m). We obtained the expression value of 1391 mature miRNAs and identified 138 differentially expressed (DE) miRNAs between high and low altitudes. Combined with tissue specificity analysis, we illustrated alterations of expression levels among altitudes and tissues, and found that there were coexisting tissue-specific and -conserved mechanisms for hypoxia acclimatization. Notably, the interplay between DE miRNA and DE target genes strongly indicated post-transcriptional regulation in the hypoxia inducible factor 1, insulin, and p53 signaling pathways, which might play significant roles in high-altitude acclimatization in domestic goats. It’s also worth noting that we experimentally confirmed miR-106a-5p to have a negative regulation effect on angiogenesis by directly targeting *FLT-1*. These results provide insight into the complicated miRNA expression patterns and regulatory mechanisms of high-altitude acclimatization in domestic goats.

## Introduction

Environmental changes impose the pressure of natural selection on vertebrates, which supports the evolution of adaptive mechanisms for survival. As a typical example of adaptive evolution in extreme environments (Richalet, 2007), high-altitude acclimatization refers to the heritable and irreversible changes in morphology (Brutsaert et al., 1999; Miles et al., 2009), physiology (Storz et al., 2009; Naeije, 2010), biochemistry (García-Hjarles, 1989; Li et al., 2010), and ethology (Mamatov et al., 2012) in highland habitants during long-term selection pressure, such as reduced oxygen availability, low ambient temperatures, and high ultraviolet radiation (UV; Brookfield and Allen, 1989; Wang et al., 2014). These changes are primarily triggered on a molecular level to modulate organic metabolism, especially in representatively hypoxia-sensitive tissues such as heart (Jarmakani et al., 1978; Wilson et al., 1993; Moromisato et al., 1996; Park et al., 2007; Chen et al., 2009), kidney (Luks et al., 2008; Dan et al., 2010; Yijiang et al., 2013), liver (Komolova and Egorov, 1985; Moromisato et al., 1996; Rong and Gastroenterology, 2009), lung (Moromisato et al., 1996; Liu, 2011; Li et al., 2012), *longissimus* muscle (Lundby et al., 2003, 2009; Murray, 2009; Levett et al., 2012; Zhang et al., 2015), and spleen (Ou et al., 1980; Kam et al., 1999; Richardson et al., 2008). Previous studies reported sufficient evidence for a molecular genetic basis of high-elevation acclimatization (Breen et al., 2008; Simonson et al., 2010; Scott et al., 2011; Bigham et al., 2013; Yang et al., 2017), and genomic patterns of genetic variation among populations and species have been widely elucidated in recent years (Cheviron and Brumfield, 2012; Mingzhou et al., 2013; Qu et al., 2013; Ai et al., 2014; Zhou et al., 2014). For instance, key erythropoiesis-related genes including *EPAS1*, *PRARA*, and *EGLN1* were found mutated in Tibetans, which were involved in hypoxia inducible factor 1 (HIF-1) signaling pathway and modulate erythropoiesis in a HIFs-centered manner. Independent or synergetic variants in these genes might be responsible for erythropoiesis responses to hypoxia in Tibetans (Simonson et al., 2010). Moreover, researchers found that the *RGCC* gene was under strong selection during high-altitude adaptation of Tibetan pig, which plays a key role in hypoxia-induced anti-angiogenesis. Interacted with *HIF-1*α and *VEGF*, preferentially selected variants in *RGCC* may inhibit the capillary growth and consequently avoid capillary leak in Tibetan pigs under long-term exposure to the hypoxic environment (Ai et al., 2014).

microRNAs (miRNAs) are a class of endogenous ∼20-nt non-coding RNAs that regulate gene expression at the post-transcriptional level via imperfect matches in their target mRNA 3’UTR regions and consequential translational repression or mRNA degradation (Bartel, 2004, 2009). Recent research on human and model animals revealed that miRNAs serve as important regulators in biological processes implicated in acclimatizations to hypoxia (Pocock, 2011), cold exposure (Al-Fageeh and Smales, 2006; Pilotte et al., 2011), and UV damage (Wan et al., 2011), which are environmental conditions associated with high altitudes (Bigham et al., 2013). For example, the miR-15 family is regulated by hypoxia and predicted to influence cardiomyocyte cell survival by regulating the expression of several pro-survival proteins (Hitoo et al., 2010; Lin et al., 2010; Small et al., 2010; Dejean et al., 2011). The UV-induced miR-211 regulates the UV-responsive cell-cycle control gene p27 and affects the proliferation potential of human prostate carcinoma cell lines (Silvia et al., 2007; Pothof et al., 2009). The well-documented hypoxia-regulated microRNA (HRM), miR-210, is directly regulated by HIF-1 (Huang et al., 2009) and participates in DNA damage response (Crosby et al., 2009), angiogenesis (Camps et al., 2008), apoptosis (Kulshreshtha et al., 2007), and mitochondrial respiration (Chan et al., 2009). However, understandings in the role of miRNA in high-altitude acclimatization have mainly focused on either human or model animals, and research on natural populations of non-model species has lagged behind.

While previous research on high-altitude goats mostly described their phenotypic characteristics, geographical distribution and living habits, molecular mechanisms behind their high-altitude acclimatizations have been poorly illustrated. As the genetic characteristics remain unclear, many important economic traits could not been fully utilized. One way for breakthrough is to study the adaptational changes of goats at small RNA transcriptome level. Therefore, we performed comparative miRNA transcriptome analysis on two populations of domestic goats, which are typical agricultural animals that reside in geographically neighboring regions at distinct altitudes (600 and 3000 m). Six hypoxia-sensitive tissues (heart, kidney, liver, lung, longissimus muscle, and spleen) were sampled from each individual and 36 small RNA libraries were constructed for sequencing.

## Results

### Data Summary

We selected three females as biological replicates from the low- and high-altitude domestic goat populations, and sampled six representative hypoxia-sensitive tissues (heart, kidney, liver, lung, *longissimus* muscle, and spleen) from each replicate. In total, 36 samples were separately used to construct small RNA libraries with an average RNA integrity number (RIN) of 7.97 (7.97 ± 0.79, *n* = 36), which were overall appropriate for small RNA-seq (Supplementary Table S1). Deep sequencing generated an average of 12.25 million (M) (12.25 ± 1.09 M, *n* = 36) raw reads for each library, and resulted in a total of 441.17 M raw reads. Through a series of quality control procedures, an average of 11.99 M (11.99 ± 0.93 M, *n* = 36) reads were retained as high-quality reads for each library, and a total of 431.51 M high-quality reads were used for miRNA identification. Consistent with the structural basis of animal miRNA, high-quality reads were mainly (79.49%) between 21 and 24-nt long, with the majority being 22-nt long (45.06%) (Supplementary Figure S1).

With stringent criteria in the miRDeep2 pipeline, a total of 909 pre-miRNAs that encode 1391 mature miRNAs were identified in all 36 libraries, including pre-miRNAs that were processed into either 5p or 3p mature forms, or both 5p and 3p mature forms. According to sequence conservation between identified mature miRNAs and annotated goat or other mammal miRNAs recorded in miRbase release 21 (Kozomara and Griffiths-Jones, 2014), 447 miRNAs sharing exactly the same mature sequence with goat annotated miRNAs were categorized into the “Known” group, whereas the 586 miRNAs with 100% sequence similarity to other mammalian miRNAs were categorized into the “Conserved” group, and the remainder, which did not match any annotated miRNA sequences, were categorized into the “Novel” group (Table 1).

**TABLE 1:** Mature miRNAs and pre-miRNAs identified in this study.

Type	Pre-miRNAs	miRNA-5p	miRNA-3p	Both ends	Mature miRNAs
Known	243	11	28	204	447
Novel	243	49	79	115	358
Conserved	423	113	147	163	586
Total	909	173	254	482	1391

To study the mechanisms that may underlie miRNA origins, we screened for overlapping miRNA precursor sequences in the genome with genomic elements of different types, including exons, introns of protein-coding genes and intergenic regions (Supplementary Figure S3). The analysis of genomic sources showed that, out of 909 pre-miRNAs, 426 (46.86%) were located in introns, followed by 345 pre-miRNAs (37.95%) in intergenic regions as the second largest category. Only three miRNA precursors (0.32%) were located in exons. Due to alternative splicing of protein-coding genes, 123 pre-miRNAs (13.53%) resided in both intronic and exonic regions of their host genes. A detailed analysis revealed that intronic regions were highly overrepresented with miRNA loci (permutation test, *P* < 0.001), which is consistent with the findings of a previous report (Berezikov, 2011).

### Global miRNA Expression Profile Analysis

**FIGURE 1:**
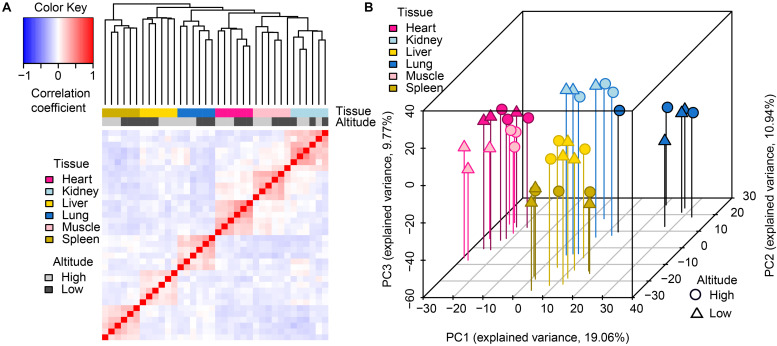
Global pattern of miRNA expression among tissues and altitudes. **(A)** Hierarchical clustering of samples using miRNA expression. Average linkage hierarchical clustering was used with the distance between samples measured by Pearson’s correlation between the vectors of z-score standardized expression values; **(B)** PCA of miRNA expression. The proportion of the variance explained by the principal components (PCs) is indicated in parentheses. The vertical lines with different colors from the plotted points dropping to the x/y plane show the separation of points based on the first and second PCs.

Through hierarchical clustering of global gene expression profiles from each sample, we found that the clustering of gene expression was tissue-dominated (Figure 1A). The samples were almost perfectly clustered by tissue and then by altitude, as directly shown by color bars above the heatmap. This expression pattern indicated that global miRNA expression varies more across tissues than between altitudes, which was consistent with the findings of a previous study (Anamaria and Henrik, 2014). Among the six tissues, the muscle and heart were more similar to each other than to other tissues, which reflects the relatively similar physiological biochemical characteristics between the two types of striated muscles (Jason et al., 2012). The clustering of biological replicates also supported experimental reliability. Results of principal components analysis (PCA) further supported these findings (Figure 1B), namely expression patterns including tissue-domination, similarity between muscle and heart, as well as the adjacent clustering of biological replicates.

### Tissue Specificity Analysis

**FIGURE 2:**
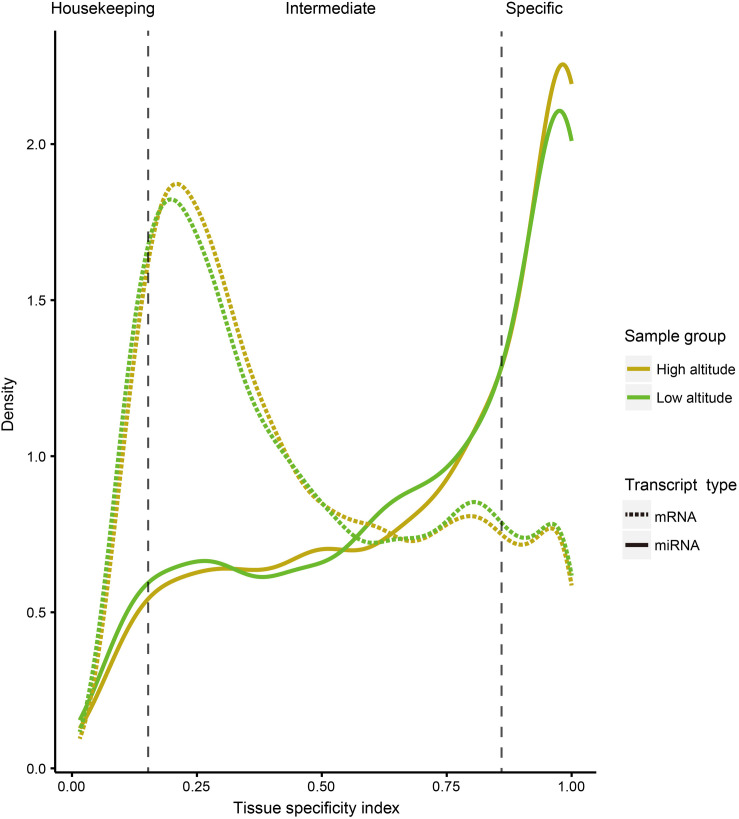
Distribution curve for TSI of miRNAs and protein-coding genes in different tissues. The vertical dotted lines correspond to the threshold originally proposed for defining housekeeping and specifically expressed transcripts with TSI values <0.15 and >0.85, respectively.

To compare tissue specificity between miRNAs and protein-coding genes, we extracted mRNA transcriptome data sets of the corresponding goat tissues from our previous study (Tang et al., 2015), and performed tissue specific index (TSI) and tissue specific score (TSS) calculations on expression profiles of both miRNA and protein-coding genes (section “Materials and Methods”). Additionally, to test whether the tissue specificity is affected by altitudes, these calculations were separately conducted based on the expression profiles of 18 samples from high- and low-altitude goat populations (Figure 2). However, these curves indicated little impact of tissue specificity by high-altitude acclimatization on both miRNAs and protein-coding genes. A sizable proportion of miRNAs (40.47%) were tissue-specific, compared with only 12.15% of protein-coding genes, as determined by analyzing expression profiles across both altitudes.

**FIGURE 3:**
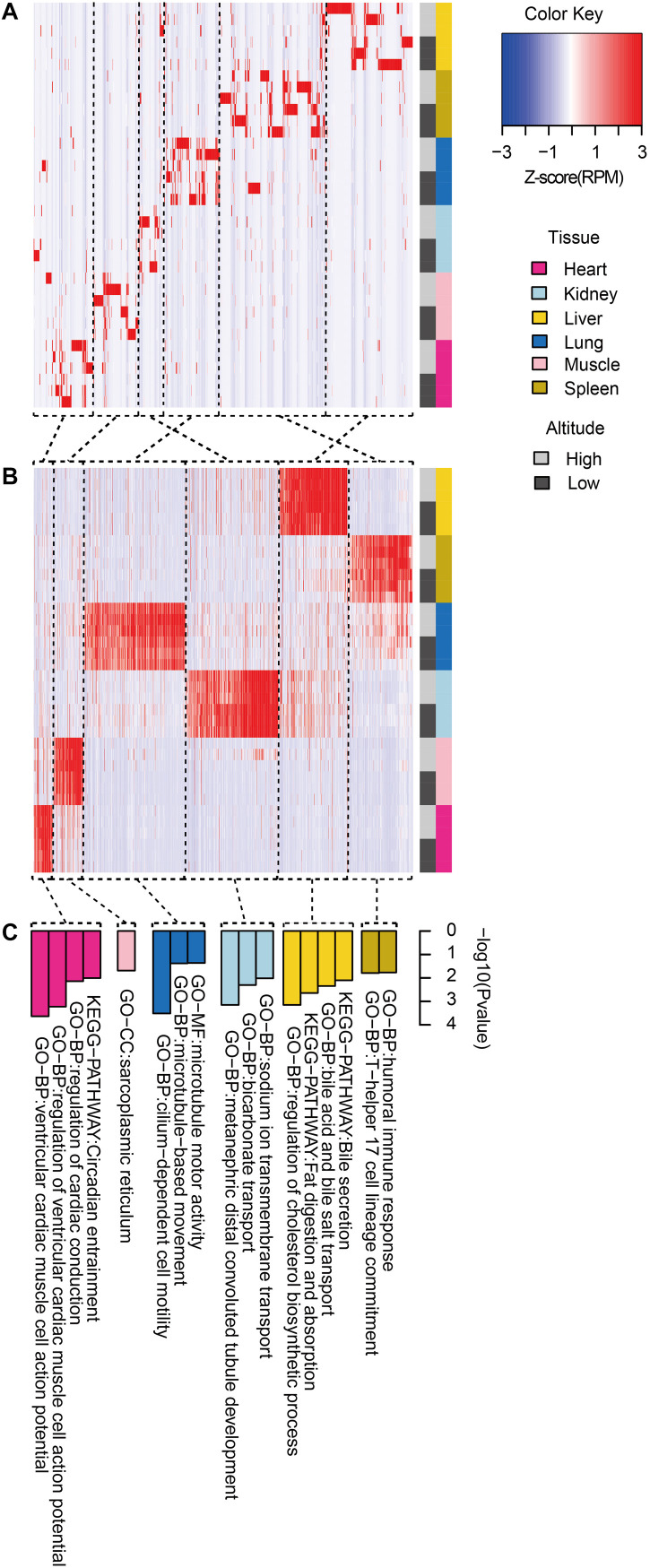
Functional pattern of tissue specificity for miRNAs and protein-coding genes. Hierarchical clustering of **(A)** miRNAs and **(B)** protein-coding genes. Average linkage hierarchical clustering was used, with the distance between transcripts measured by Pearson’s correlation between the vectors of the z-score standardized expression values. **(C)** Functional enrichment of tissue-specific genes targeted by tissue-specific miRNAs within corresponding tissues. Representative GO terms and KEGG pathways.

We then clustered tissue-specific miRNAs (Figure 3A) and genes (Figure 3B) using single-linkage clustering with expression and annotated each cluster with enriched functions of tissue-specific genes targeted by tissue-specific miRNAs within corresponding tissues (Figure 3C). Indeed, clusters of tissue-specific miRNAs and genes were enriched in functional categories specific to certain tissue. For example, target genes of the heart-specific cluster were significantly enriched in functional terms such as regulation of cardiac conduction (three genes, *P* = 5.89 × 10^–4^) and ventricular cardiac muscle cell action potential (two genes, *P* = 9.79 × 10^–3^). Muscle-specific target genes were enriched in sarcoplasmic reticulum (two genes, *P* = 2.03 × 10^–2^). Kidney-related terms, such as metanephric collecting duct development (three genes, *P* = 1.15 × 10^–4^) and sodium ion transmembrane transport (four genes, *P* = 6.94 × 10^–4^), were enriched in kidney-specific target genes. Microtubule motor activity (five genes, *P* = 3.02 × 10^–4^) and cilium-dependent cell motility (three genes, *P* = 4.15 × 10^–2^) were detected as enriched Gene Ontology (GO) terms in lung-specific genes. Additionally, over-representation of humoral immune response (three genes, *P* = 1.60 × 10^–2^) in spleen-specific genes and bile secretion (four genes, *P* = 6.98 × 10^–4^) in liver-specific genes were also found.

### Comparison of miRNAs Between the High- and Low-Altitude Populations

To explore the transcriptional changes of miRNAs induced by acclimatization of goats to high-altitude stress, we performed comparisons between high- and low-altitude goat populations within each tissue. In total, we detected 138 differentially expressed mature miRNAs between high- and low-altitude populations (heart, 37; kidney, 20; liver, 40; lung, 26; muscle, 47; spleen, 37) (Table 2).

**TABLE 2:** Number of DE miRNAs between high- and low-altitude populations within each tissue.

Tissues	Heart	Lung	Spleen	Muscle	Liver	Kidney	Total
Number of DE miRNAs	37	26	37	47	40	20	138

**FIGURE 4:**
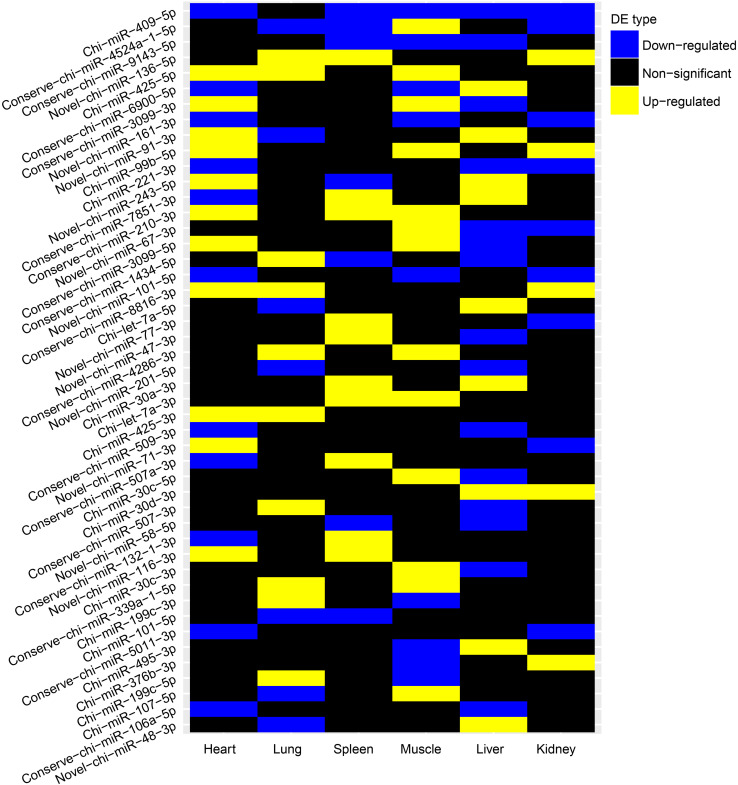
Pairwise DE miRNAs between altitudes of each tissue. High altitude vs low altitude.

We further evaluated whether these differentially expressed (DE) miRNAs showed similar expression alteration patterns among the six tissues, because of the strong natural selection pressure on high-altitude goat populations. Tissue-specific analysis of DE miRNAs showed that miRNAs with expression changes between altitudes had a lower proportion of tissue-specific miRNAs than those that were not differentially expressed (Supplementary Figure S4). Evaluation of the overlap of expression alterations across tissues revealed that most of the 138 DE miRNAs (*n* = 91) underwent expression changes with no overlap among tissues, whereas the rest (*n* = 47) showed consistent up- or down-regulation among up to five tissues (Table 3). Interestingly, miRNAs differentially expressed in only one tissue were also significantly (Chi-squared test, *P* = 9.55 × 10^–6^) more tissue-specific (41/91) than those with expression alterations in more than one tissue (3/47) (Supplementary Figure S5). It is worth noting that those 41 tissue-specific non-overlapping DE miRNAs were generally enriched in tissue-related functions, such as calmodulin binding (nine genes, *P* = 7.20 × 10^–3^) in heart, regulation of ion transmembrane transport (five genes, *P* = 5.36 × 10^–3^) in kidney, and positive regulation of fibroblast proliferation (seven genes, *P* = 4.03 × 10^–4^) in muscle (Supplementary Table S2). Alternatively, the overlapping DE miRNAs functioned in more widespread processes in high-altitude acclimatization (Figure 4); for example, miR-409-5p was consistently down-regulated in five tissues (heart, kidney, liver, muscle, and spleen) of the high-altitude goat population, and its target genes were significantly enriched in hypoxia-related functions, such as positive regulation of angiogenesis and apoptosis.

**TABLE 3:** Number of DE miRNAs between high- and low-altitude populations overlapping among tissues.

Number of tissues	1	2	3	4	5	6
Number of overlapping DE miRNAs	91	28	17	1	1	0

### DE miRNAs Involved in High-Altitude Acclimatization

To illustrate the potential functions of identified DE miRNAs on a larger scale, we performed target prediction on DE miRNAs and only maintained miRNA–mRNA pairs with negative correlation coefficients as high-confidence target pairs for further analysis. In particular, a considerable portion of high-confidence target genes were detected with known or potential roles in high-altitude-related responses, such as apoptosis, angiogenesis, DNA damage repair, erythropoiesis, and energy metabolism, which are highly related to high-altitude acclimatization according to previous studies (Supplementary Table S3).

**FIGURE 5:**
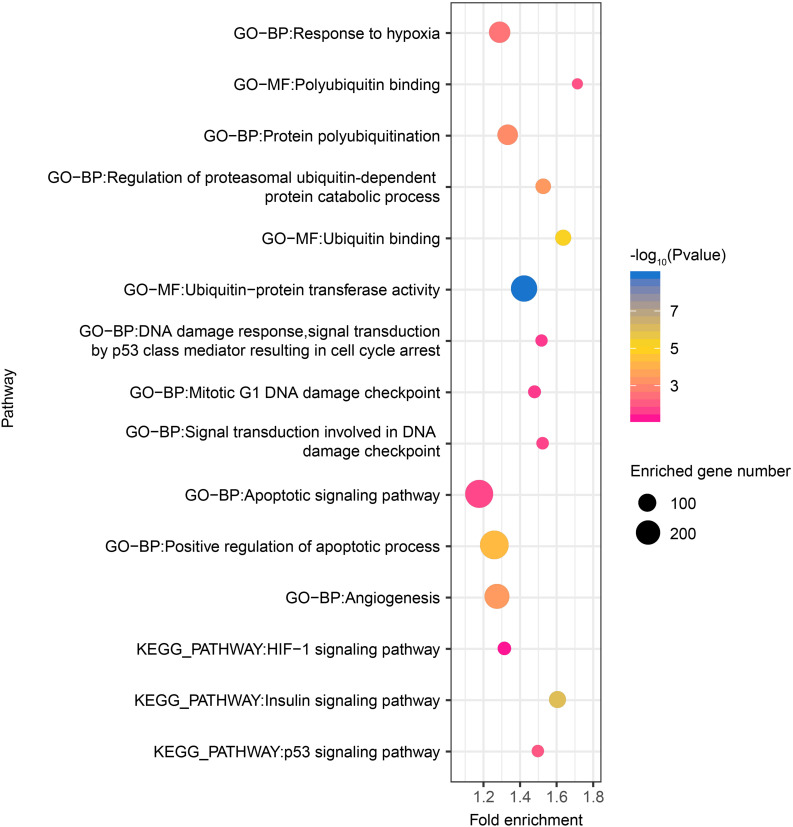
Hypoxia-related functional gene categories enriched by target genes of DE miRNAs among all six tissues between high- and low-altitude populations. The *P* value was calculated using the Benjamini-corrected modified Fisher’s exact test. BP, biological process; MF, molecular function; CC: cellular component.

Thereafter, as revealed by functional enrichment analysis of high-confidence target genes, the majority of target genes might have known or potential roles in hypoxia response among all six tissues (Figure 5). For example, target genes were significantly enriched in protein-ubiquitination related categories, including ubiquitin binding (75 genes, *P* = 5.5 × 10^–6^), ubiquitin-protein transferase activity (238 genes, *P* = 1.8 × 10^–9^), and regulation of proteasomal ubiquitin-dependent protein catabolic process (69 genes, *P* = 3.7 × 10^–4^), which serves as a critical component of hypoxia response. Additionally, target genes were also significantly enriched in categories related to DNA damage repair, such as mitotic G1 DNA damage checkpoint (43 genes, *P* = 2.7 × 10^–2^) and signal transduction involved in DNA damage checkpoint (40 genes, *P* = 2.0 × 10^–2^). Moreover, functional enrichment in positive regulation of apoptotic process (288 genes, *P* = 5.25 × 10^–5^) and angiogenesis (211 genes, *P* = 3.63 × 10^–4^) were also found in target genes shared by all six tissues, as were pathways such as HIF-1 (49 genes, *P* = 5.66 × 10^–2^), p53 (39 genes, *P* = 1.00 × 10^–2^), and insulin signaling pathways (86 genes, *P* = 1.09 × 10^–6^).

**FIGURE 6:**
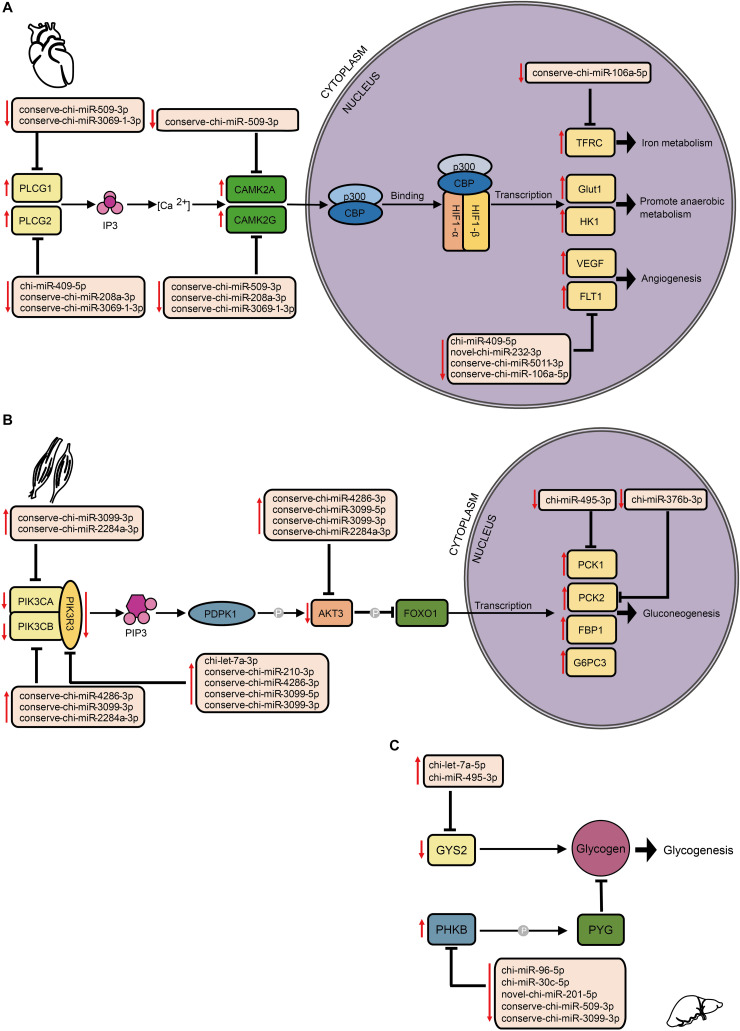
Schematic representation of hypoxia-related signaling pathways enriched by target genes of DE miRNAs in different tissues, including **(A)** the HIF-1 signaling pathway in heart, and the insulin signaling pathway in **(B)** muscle and **(C)** liver. The up and down arrows in red indicate up- and down-regulation, respectively, of DE miRNAs or genes. The clip art of tissues were created on our own.

By comparing mRNA expression alterations of these target genes between altitudes, we found more reliable evidence for acclimatization based on miRNA–mRNA interactions in hypoxia-related pathways. Our findings mainly focused on the interplay between DE miRNA and target genes, especially in the HIF-1 signaling pathway (Figure 6A). By collectively analyzing all six tissues, we found 102 pairs that consisted of 37 DE miRNAs with 31 target genes that were previously confirmed to be differentially expressed involved in both upstream and downstream of the HIF-1 signaling pathway (Supplementary Table S4). In heart, genes encoding both isoforms of phospholipase Cγ (PLCγ), *PLCG1*, and *PLCG2* (Guoping et al., 2012), were found to be up-regulated by decrease of the DE miRNAs conserve-chi-miR-509-3p and conserve-chi-miR-3069-1-3p (*PLCG1*), and chi-miR-409-5p, conserve-chi-miR-3069-1-3p, and conserve-chi-miR-208a-3p (*PLCG2*). Genes that encode isoforms of the downstream Ca2t/calmodulin kinase II (CaMKII), *CAMK2A* and *CAMK2D* (Swulius and Waxham, 2008), were also up-regulated because of reduced expression of the DE miRNAs conserve-chi-miR-509-3p (*CAMK2A*) and conserve-chi-miR-509-3p, conserve-chi-miR-208a-3p, and conserve-chi-miR-3069-1-3p (*CAMK2D*) (Figure 6A). As expected, mRNA expression levels of HIF-1 target genes, including *VEGFA*, *FLT1*, *Glut1*, *HK1*, and *TFRC*, that were involved in angiogenesis, anaerobic metabolism, and iron metabolism were significantly up-regulated, and the transcription of *FLT1* and *TFRC* were further activated by decrease of novel-chi-miR-232-3p, conserve-chi-miR-5011-3p, chi-miR-409-5p, and conserve-chi-miR-106a-5p (Figure 6A).

In the insulin signaling pathway, we detected 103 pairs of DE miRNA–mRNA (Supplementary Table S4). In muscle, we found eight DE miRNAs for which their target genes correspondingly exhibited negative regulation. Upstream of the transcription factor FOXO1, genes encoding PI3K subunits and Akt were modulated down. Alternatively, the genes that encode PCK1 and PCK2 were up-regulated (Figure 6B). In liver, the expression levels of chi-miR-495-3p and chi-let-7a-5p significantly increased, and the target gene *GYS2* had decreased expression. Moreover, the gene encoding phosphorylase kinase regulatory subunit beta (PHKB) was simultaneously up-regulated with the down-regulation of novel-chi-miR-201-5p, conserve-chi-miR-509-3p, chi-miR-96-5p, chi-miR-30c-5p, and conserve-chi-miR-3099-3p.

**FIGURE 7:**
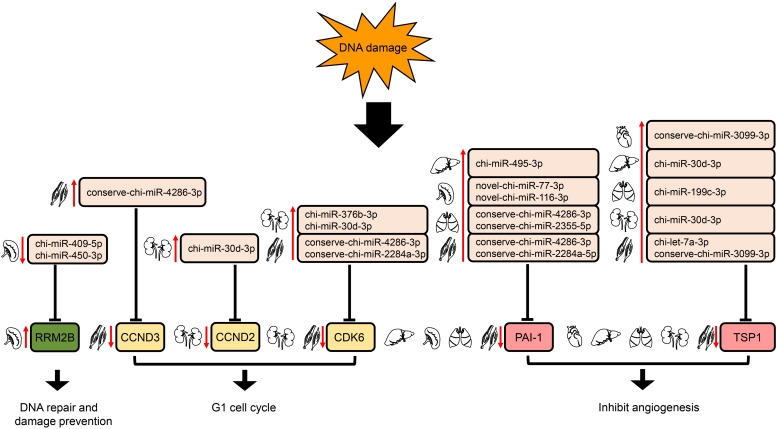
Regulation of genes targeted by DE miRNAs enriched in the downstream of p53 signaling pathway. The up- and down-arrows in red mean up- and down-regulation of DE miRNAs or genes, respectively. The clip art of tissues were created on our own.

In the p53 signaling pathway, we found 69 pairs of DE miRNA–mRNA (Supplementary Table S4). Interestingly, *PAI-1* and *TSP1* were down-regulated, whereas their corresponding target miRNAs were up-regulated in liver, lung, and muscle. The down- and up-regulation of *TSP1* and its target miRNA were also detected in heart and kidney. In addition, chi-miR-409-5p and chi-miR-450-3p in spleen were down-regulated, with increased expression of their target gene, *RRM2B*. Furthermore, up-regulation of several DE miRNAs was found along with down-regulation of their target genes, *CDK6* in both kidney and muscle and *CCND2* (kidney)/*CCND3* (muscle) (Figure 7).

As confirmed by qRT-PCR, the fold change of relative expression between low- and high-altitude were found significantly correlated with the fold change of normalized read counts in DE miRNAs (9/11, Supplementary Table S5), especially for those that are likely to play a crucial role in high-altitude acclimatization-related pathways. Next, we selected nine candidate mRNA–miRNA pairs and performed the dual luciferase reporter assays to validate the potential relationship of these pairs. As shown in Supplementary Figure S6, eight miRNAs significantly repressed luciferase activity in HeLa cells transfected with the 3’-UTR reporter, which demonstrated the robustness of mRNA–miRNA pairs in this study. Thus, these results provide robust evidence for high-altitude acclimatizations in domestic goats based on miRNA expression alterations.

### MiR-106a-5p Had a Negative Regulation Effect on Angiogenesis

**FIGURE 8:**
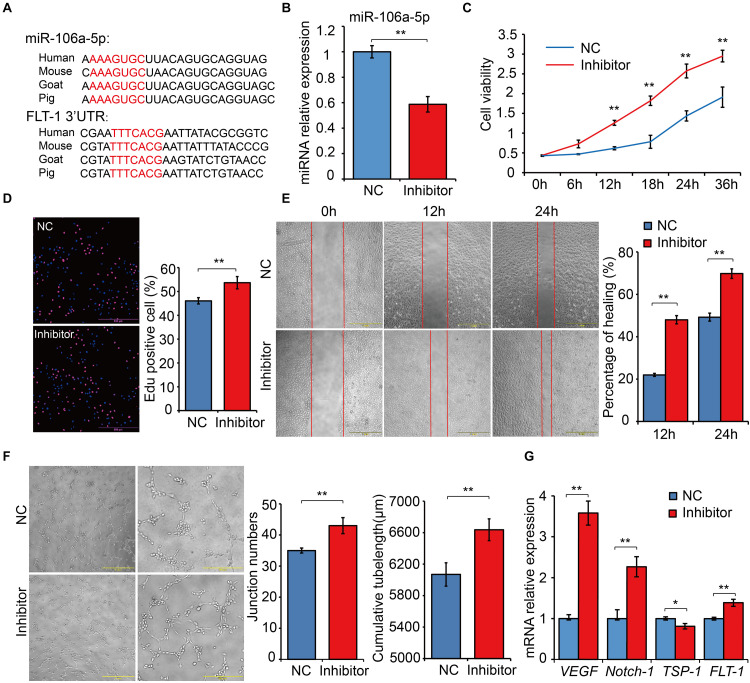
Functional experiments of miR-106a-5p regulation effect on angiogenesis. **(A)** Sequence alignment analysis of miR-106a-5p and the 3’-UTR of *FLT-1*. **(B)** miR-106a-5p inhibition efficiency analysis using qRT-PCR. miR-106a-5p inhibitor or NC was transfected into HUVECs, cell viability **(C)**, proliferation **(D)**, migration **(E)** and angiogenesis **(F)** were assessed by CCK-8 detection, Edu staining, scratch assay and tube formation experiments, respectively. **(G)** qRT-PCR quantification of pro-angiogenesis genes, including *VEGF* and *Notch-1*, and anti-angiogenesis gene (*TSP-1*) and the target gene of miR-106a-5p (*FLT-1*). At least three independent experiments were repeated three times. All values are presented as mean ± standard deviation (SD).

Based on the results of dual luciferase assays, we noticed that *FLT-1* was the direct target gene of miR-106a-5p, and *FLT-1*, also named *VEGFR1*, were reported to be involved in angiogenesis (Hiratsuka et al., 2001; Shibuya, 2006). Thus, miR-106a-5p was selected as a representative for exploring the biological function of DE miRNAs involved in high-altitude acclimatization. Sequence alignment analysis showed that miR-106a-5p manifested high similarity especially in seed sequence among several representative species (Figure 8A). Meanwhile, the 3’-UTR of *FLT-1* containing the miR-106a-5p binding site were also conserved among these species (Figure 8A). Given lack of commercial cell line in goat, we performed miRNA function analysis using the human umbilical venous endothelial cells (HUVECs), a widely used cell line for angiogenesis research. Effective inhibition of miR-106a-5p was confirmed using qRT-PCR (*P* < 0.01, Figure 8B). As the angiogenesis involves many key processes, including proliferation, migration and tube formation (Wang et al., 2011), we thus assessed them by CCK-8 detection, Edu staining, scratch assay and tube formation experiments. These results showed that miR-106a-5p downregulation promoted HUVECs proliferation (*P* < 0.01, Figures 8C,D), enhanced migration ability (*P* < 0.01, Figure 8E), and increased novel cellular junctions and tube-length (*P* < 0.01, Figure 8F), compared with NC. Meanwhile, the expression level of pro-angiogenesis genes including *VEGF, FLT-1* and *Notch-1* significantly increased, and that of anti-angiogenesis gene *TSP1* decreased (*P* < 0.01, Figure 8G). These results indicated that miR-106a-5p had a negative regulation effect on angiogenesis.

## Discussion

To date, no studies have illustrated the role of goat miRNA in high-altitude acclimatization. Hence, to the best of our knowledge, this is the first report to compare goat miRNA profiles between altitudes and among multiple tissues.

As a result of tissue specificity analysis for miRNAs and protein-coding genes, miRNAs exhibited more tissue specificity than protein-coding genes in goats (Figure 2), which is consistent with the findings of a previous study on human (Ludwig et al., 2016). Further analysis for tissue-specific miRNA–mRNA target pairs showed clustering of both tissue-specific miRNA (Figure 3A) and mRNA (Figure 3B), which indicated the similar expression pattern of tissue-specific transcripts in each tissue. In particular, enrichment in tissue-related functional categories were found in clusters of tissue-specific genes targeted by tissue-specific miRNAs (Figure 3C). Therefore, the potential regulation of tissue-specific protein-coding genes by tissue-specific miRNAs may play a crucial role in tissue function maintenance.

As a result of miRNA differential expression, muscle exhibited the highest number of DE miRNAs among the six tissues. This highly dramatic transcriptional change in the muscle reflects that, among the six tissues, skeletal muscle cells were most sensitive to hypoxia and most closely associated with high-altitude acclimatization (Howald and Hoppeler, 2003; Murray, 2009; Levett et al., 2012).

Combining expression alterations and tissue specificity of miRNAs, we found that miRNAs with expression changes between altitudes were clearly less tissue-specific than those that were not differentially expressed (Supplementary Figure S4). Despite of this, most of the DE miRNAs underwent expression changes with no overlap among tissues, whereas the rest showed up- or down-regulation in multiple tissues (Table 4). Interestingly, miRNAs differentially expressed in only one tissue were also more tissue-specific than those with expression alterations in more than one tissue (Supplementary Figure S5). Further more, we found that tissue-specific non-overlapping DE miRNAs were generally enriched in tissue-related functions, whereas the overlapping DE miRNAs functioned in more widespread processes in high-altitude acclimatization (Figure 4). With consistent up/down-regulation in multiple tissues, the tumor suppressor miR-425 is able to repress the PI3K-Akt pathway by targeting IGF-1 (Liu et al., 2015). The key mediator of mTOR kinase, miR-99b, contributes to radiation-induced mTOR up-regulation, which plays a critical role in radio-resistance (Wei et al., 2013). miR-221 has the ability to block endothelial cell migration, proliferation, and angiogenesis *in vitro* by targeting c-Kit and indirectly regulating expression of eNOS (Angelika et al., 2008). miR-210 serves as the micromanager of the hypoxia pathway and regulates many hypoxia-related processes, such as angiogenesis, cell cycle regulation, and DNA damage repair (Huang et al., 2010). Taken together, the expression alterations and tissue-specificity of miRNAs among tissues suggested not only the potential tissue-specific mechanism to maintain normal tissue functions under hypoxia, but also the tissue-conserved miRNA acclimatization to high altitude.

**TABLE 4:** Summary and phenotypic characteristics of sampled goats.

Population description	Breed	Location	Altitude (m)	Age (year)	Body weight (kg)	Forms of livestock rearing
Tibetan goat (high-altitude)	Tibetan goat	Songpan prefecture, Tibetan autonomous region, China	3120	Adult (3.21 ± 0.09, *n* = 3)	25.16 ± 3.39, *n* = 3	Stocking/nomadism
Indigenous goat (low-altitude)	Chuandong White goat	Ya’an city, Sichuan Province, China	614	Adult (3.16 ± 0.08, *n* = 3)	41.24 ± 3.72, *n* = 3	Home rearing

Furthermore, literature search on high-confidence target genes of DE miRNAs indicated their roles in high-altitude-related biological processes (Supplementary Table S3). For example, the energy metabolism-related genes *USP1*, *JAK2*, *MTPN*, and *PDPK1* were predicted targets of the DE miRNA conserve-chi-miR-375-3p in this study, which is consistent with previous research (Poy et al., 2004, 2009; El Ouaamari et al., 2008); similarly, *IGF1R*, *INSR*, and *IRS2* were targeted by chi-let-7a-3p (Sun et al., 2009; Zhu et al., 2011). This finding is also well supported by functional enrichment of target genes (Figure 5). For instance, three classes of target genes were found significantly enriched in protein-ubiquitination related categories. Since HIF-1α protein degradation in hypoxia is ubiquitination-dependent, and E3 ubiquitin ligases may participate in this degradation pathway (Ivan et al., 2001; Jaakkola et al., 2001; Anna et al., 2004; Nakayama et al., 2009; Wang et al., 2016), these target genes are likely to participate in hypoxia response. Analogously, target genes were also found enriched in categories related to DNA damage repair. As it is common knowledge that high-altitude exposure results in decreased oxygen pressure and increased UV radiation, which lead to DNA damage (Dosek et al., 2007), we speculate that Tibetan goats might have evolved strong abilities to resist DNA damage caused by hypoxia and intense UV radiation during long-term acclimatization to the extreme environmental conditions in the Tibetan Plateau.

Through comparisons of miRNA–mRNA expression alterations between altitudes, we found considerable evidence for acclimatization based on miRNA–mRNA interactions in hypoxia-related pathways, including HIF-1, p53, and insulin signaling pathways. HIF-1 is a basic transcription factor that is expressed in all metazoan organisms and consists of HIF-1α and HIF-1β subunits, and it functions as a master regulator of oxygen homeostasis (Semenza, 2007). Under hypoxic conditions, HIF-1 regulates the transcription of numerous hypoxia-response genes involved in angiogenesis (Fraisl et al., 2009) and energy metabolism (Ke and Costa, 2006) by binding to hypoxia-response elements. Moreover, during high-altitude hypoxia, ROS production by the mitochondrial electron transport system can increase (Mohanraj et al., 1998), while increased levels of ROS have been shown to activate PLCγ (González-Pacheco et al., 2002), which generates inositol 1, 4, 5-trisphosphate (IP3). Activated IP3 receptors can lead to elevations in Ca^2+^ via mobilization of intracellular Ca^2+^ stores (Yuan et al., 2010), which activates CaMK (Ying-Jie et al., 2010) and thereby phosphorylates the HIF-1 co-activators p300 and CREB-binding protein (Hardingham and Arnold, 2001) to form an effective transcriptional complex that can regulate the transcription of target hypoxia-response genes (Azimi, 2018). In this study, the down-regulation of conserve-chi-miR-509-3p, conserve-chi-miR-3069-1-3p, chi-miR-409-5p and conserve-chi-miR-208a-3p in heart were possibly responsible for the expression elevation of genes encoding PLCγ and CaMK, which promote the transcriptional activity of HIF-1. Downstream of HIF-1 transcriptional complex, mRNA expression levels of several HIF-1 target genes that were involved in angiogenesis, anaerobic metabolism, and iron metabolism were found significantly up-regulated (Figure 6A). Notably, we confirmed that *FLT-1* was a direct target gene of miR-106a-5p by dual luciferase reporter assay. FLT-1 is the receptor of VEGF (Hiratsuka et al., 2001; Shibuya, 2006) and plays important roles in endothelium angiogenesis (Wang et al., 2011). Functionally, miR-106a-5p downregulation could promote angiogenesis through promoting proliferation, enhancing migration ability, and increasing tube formation of endothelial cells by mitigating its inhibition effect on *FLT-1*, which further elucidated the potential mechanism of miRNA-mediated regulations in angiogenesis. Therefore, the expression alterations of miRNAs might play an important role in high-altitude acclimatization through the HIF-1 signaling pathway.

Insulin functions as the primary hormone involved in glucose homeostasis and the stimulation of glucose transport through the insulin signaling pathway (Saltiel and Kahn, 2001), which can be inhibited and switch to a state of insulin resistance under hypoxia (Claire et al., 2009). As previously reported, PI3K and Akt could inhibit the transcription factor FOXO1, and PCK1 and PCK2 are the key enzymes in gluconeogenesis. In muscle, the down-regulation of genes encoding PI3K subunits and Akt and the up-regulation of genes that encode PCK1 and PCK2 (Figure 6B) indicated enhanced glucose production via gluconeogenesis in response to hypoxia. *GYS2* encodes the key isozyme of glycogenesis, whereas PHKB phosphorylates and activates glycogen phosphorylase (PYG) (Tung et al., 1984). Because PYG catalyzes the rate-limiting step in glycogenolysis and breaks up glycogen into glucose subunits (Newgard et al., 1989), the down-regulation of *GYS2* and up-regulation of *PHKB* by corresponding DE miRNAs in liver (Figure 6C) suggested inhibited liver glycogenesis at high-altitude. Hence, DE miRNAs might participated in the regulation of glycometabolism via the insulin signaling pathway and help with energy expenditure under the severe environment at high altitude.

The p53 pathway is composed of hundreds of genes and their products that respond to a wide variety of stress signals, such as DNA damage and hypoxia (Levine et al., 2006). p53 activation triggers the transcription of numerous genes involved in cell cycle arrest, apoptosis, DNA repair, and anti-angiogenesis (Harris and Levine, 2005). In liver, lung, and muscle, the angiogenesis inhibitors (Rodriguez-Manzaneque et al., 2001; Skrzypczakjankun, 2001) *PAI-1* and *TSP1* were modulated down due to the up-regulation of their corresponding target miRNAs. Whereas in heart and kidney, *TSP1* was down-regulated by the expression elevation of its target miRNA (Figure 7). Moreover, down-regulation of chi-miR-409-5p and chi-miR-450-3p in spleen likely increased expression of their target gene, *RRM2B*; this gene encodes the protein p53R2, which is necessary for DNA repair. In addition, up-regulation of several DE miRNAs might repress their target genes, *CDK6* in both kidney and muscle and *CCND2* (kidney)/*CCND3* (muscle) (Figure 7); these genes encode the key proteins in G1/S checkpoint function (Pietenpol and Stewart, 2002). Therefore, DE miRNAs involved in the p53 signaling pathway might play a significant role in responses to high-altitude environmental stress.

Despite the evidences we found in regulatory mechanisms of high-altitude acclimatization in domestic goats, we must admit the limitation that breed differences were not fully excluded in this comparative miRNA transcriptome analysis. It is of great interest to conduct the experimental design of moving the indigenous goat to high altitude for years and to better illustrate the mechanisms of high-altitude acclimatization on the same breed level.

Overall, based on our attractive goat transcriptomic resource, future work could be done on both *in silico* and experimental sides to improve our knowledge on high-altitude acclimatizations in goat populations: to further illustrate relationships among high-altitude acclimatization-related signaling pathways (Figures 6, 7) by conducting network analysis on involved miRNA–mRNA interactions, and to experimentally validate functions of highlighted miRNAs whose target genes has already been validated by our luciferase reporter assays (Supplementary Figure S6). Accompanied by the continuous development of goat genome sequence resources, fundamental questions of evolutionary adaptation in goats could be addressed in a more comprehensive way, by integrating phenotypic, population genomic and transcriptomic data.

## Conclusion

This study presents a comprehensive and systematic survey of miRNA transcriptome in goat populations at high- and low-altitude, shedding light on the complicated miRNA expression patterns and associated regulatory mechanisms of high-altitude acclimatization in domestic goats. We expanded annotation of goat miRNAs to more than three times as many as the miRBase annotation using small RNA-seq data. Combination analysis of expression alterations and tissue specificity of miRNAs suggested potential coexisting tissue-specific and -conserved mechanisms for hypoxia acclimatization. Integrated miRNA-mRNA expression profiles and functional enrichment further provided evidence of miRNA involvements in post-transcriptional regulation through HIF-1, insulin, and p53 signaling pathways, which might promote anaerobic metabolism, angiogenesis, gluconeogenesis, DNA damage repair and inhibit glycogenesis. In addition, we experimentally confirmed miR-106a-5p to have a negative regulation effect on angiogenesis by directly targeting *FLT-1*. Taken together, these findings support the viewpoint that miRNAs could play significant roles in high-altitude acclimatization of domestic goats. Our study may not only accelerate research into goats as natural models for studying high-altitude acclimatization, but also provide valuable theoretical underpinnings for further utilization of genetic resources in plateau regions.

## Materials and Methods

### Animals and Samples Collection

To control breed differences which might contribute to overall observed differences, we selected goat breeds under the following rules: (1) Located in geographically close regions within 300 km straight-line distance and (2) Without significant geographic isolation. Following these rules, we selected the indigenous goat and Tibetan goat residing at distinct altitudes in southwest China (600 and 3000 m) for comparative analysis, between which the straight-line distance was 299 km. Three adult females (∼3 years old) from each of the indigenous goat and Tibetan goat populations residing at distinct altitudes in southwest China (600 and 3000 m) were used in this study (Table 4). For the home-reared indigenous goats, we selected the individuals unrelated within three generations according to the pedigree; for the stocking Tibetan goats, we randomly selected three female individuals. All the animals were provided by local farm. To control the diet differences between rearing modes of home rearing and stocking/nomadism, we fed the home-rearing Indigenous goat with natural pasture grass instead of artificially added compound feeds. Animals were fed with free access to food and water, and killed humanely to ameliorate suffering by putting them under deep anesthesia with intravenous sodium pentobarbital (30 mg/kg body weight) before slaughter. Six typical hypoxia-sensitive tissues (heart, kidney, liver, lung, *longissimus* muscle, and spleen) were rapidly excised from the carcass, immediately frozen in liquid nitrogen, and then stored at −80°C until RNA isolation.

Research procedures involving animals were performed according to the Regulations for the Administration of Affairs Concerning Experimental Animals (Ministry of Science and Technology, China, revised in June 2004) and approved by the Institutional Animal Care and Use Committee in College of Animal Science and Technology, Sichuan Agricultural University, Sichuan, China under permit No. DKY-S20163658.

### RNA Isolation, Small RNA Library Preparation and Sequencing

Total RNA was extracted using Trizol reagent (Ambion, United States) according to the manufacturer’s protocols. Small RNA libraries were constructed using the Illumina TruSeq Small RNA Sample Prep kit. Libraries were assessed using the Agilent 2200 TapeStation and sequenced on the Illumina HiSeq 2500 platforms. Incipient bioinformatics analysis (base calling) was performed with CASAVA 1.8 (Illumina) to generate raw reads (in FASTQ form).

### Data Downloading and Processing for mRNA Transcriptome

To elucidate the effects of miRNA on downstream expression, we downloaded 36 additional transcriptome data sets of the exactly corresponding goat samples from our previous study (Tang et al., 2015) stored in the Gene Expression Omnibus (GEO) under accession code GSE66242. After a strict quality control, high-quality reads obtained were mapped to the representative goat genome (assembly ARS1) using hisat version 2.0.5 (Kim et al., 2015). Stringtie version 1.3.3 was used to quantify gene expression and obtain FPKM (denoted as fragments per kilobase of exon per million fragments mapped) expression values (Mihaela et al., 2015). We further used Cuffdiff 2 (Cole et al., 2013) to detect differentially expressed genes (DEGs) between population pairs from distinct altitudes in the six tissues. We defined genes with |log_2_ (fold change)| ≥ 1, *P* value <0.05 and adjusted *P* value <0.1 as DEGs. Differentially expressed genes with a log_2_ (fold change) < 0 were defined as “up-regulated” and those with a log_2_ (fold change) > 0 were defined as “down-regulated” at high altitude.

### Read Mapping and miRNA Identification

Raw reads was subjected to a series of stringent filters (such as removing low quality-reads, repeated sequences and adaptor sequences). Filtered high-quality sequences were then mapped to goat reference genome (assembly ARS1) with stringent criteria (0 mismatch in the whole length) using Bowtie (Langmead, 2010). The number of mappable reads were similar between high and low altitudes (Supplementary Figure S2); therefore, the miRNA libraries had unbiased sizes for further analysis. Next, mappable reads were submitted to miRDeep version 2.0.0.7 (Friedlander et al., 2012) to detect miRNAs with default parameters, while mature miRNA sequences of goat and all other annotated mammalian species in miRbase release 21 (Kozomara and Griffiths-Jones, 2014) were selected as reference. miRNAs with read count no less than 3 in at least one sample were remained for further analysis. Read counts were normalized by the total count of mappable reads of each sample, also known as RPM, for unbiased comparisons among samples. Normalized expressions were further standardized by computing standard scores (also known as z-scores) for cluster and PCA (Wichern and Johnson, 1982; Everitt and Hothorn, 2011).

### Qualitative and Quantitative Measure for Tissue Specificity

To evaluate the variability of expression patterns, we brought in two measures, TSI (Ludwig et al., 2016) and TSS (Cabili et al., 2011), which respectively indicate whether a transcript is tissue-specific and to which tissue this transcript is most tissue-specific.

The TSI is a quantitative measure for the specificity of a transcript in different tissues. The values range from 0 to 1. The higher a TSI, the more tissue-specific a transcript is. In this study, we defined a transcript with a TSI < 0.15 as “housekeeping” and >0.85 as “tissue-specific.”

The TSS is an entropy-based measure that quantifies the similarity between a transcript’s expression pattern and another predefined pattern that represent an extreme case in which a transcript is expressed in only one tissue. For each transcript, a TSS was calculated with respect to each tissue and the tissue with the maximal TSS was considered specific (e.g., heart-specific) if the TSI of this transcript is larger than 0.85.

### Identification of DE miRNAs

To compare the expression levels of miRNA transcriptome between low-altitude and Tibetan goat, we identified DE miRNAs using edgeR package (Robinson et al., 2010; Mccarthy et al., 2012). Read counts were loaded into edgeR and normalized using the built-in trimmed mean of M-values (TMM) algorithm. miRNAs with a |log_2_ (fold change)| ≥ 1.5 and a Benjamini Hochberg FDR < 0.05 between altitudes were defined as DE miRNAs. DE miRNAs with a log_2_ (fold change) < 0 were defined as “up-regulated” and those with a log_2_ (fold change) > 0 were defined as “down-regulated” at high altitude.

### Target Prediction and Functional Annotation of miRNAs

We applied TargetScan version 7.0 to DE miRNAs and 3’UTR sequences extracted from goat genome for target predications (Agarwal et al., 2015). Thereafter, we calculated Spearman’s correlations for expression levels of every miRNA-mRNA candidate target pair and maintained pairs with negative correlation coefficients as high-confidence target genes. The enrichment of GO and Kyoto Encyclopedia of Genes and Genomes (KEGG) pathway by target genes were performed using DAVID web-accessible program (Da et al., 2009) (Benjamini adjusted *P* value ≤0.05).

### Quantitative Real-Time PCR Validation

To validate gene expression, quantitative real-time PCR (qRT-PCR) was conducted on 11 miRNAs in tissue samples from high- and low-altitude goat individuals, 1 miRNA in HUVECs, and 4 mRNAs in HUVECs. miRNAs and mRNAs were reverse transcribed using a Mir-X^TM^ miRNA First Strand Synthesis Kit (Takara, Dalian, China) and PrimeScript RT reagent Kit with gDNA Eraser (Takara, Dalian, China), respectively. Quantitative real-time PCR was performed using a SYBR Premix Ex Taq kit (Takara, Dalian, China) on the CFX96TM Real-Time PCR Detection System (Bio-Rad, CA, United States). *U6* and *GAPDH* were simultaneously used as endogenous control genes for miRNAs and mRNAs, respectively. And primer sequences for qRT-PCR are shown in Supplementary Table S6. A negative control (NC) was introduced into all measurements (no cDNA template), and each RNA sample was analyzed in triplicate. We computed relative expression levels of objective miRNAs with the 2^–ΔΔCt^ method. To insure the accuracy of the 2^–ΔΔCt^ results, we carried out the optimization of annealing temperature by thermal gradient and evaluation of amplification efficiency in CFX96TM Real-Time PCR Detection System (Bio-Rad, CA, United States), and all wells used in the subsequent analysis were met 0.95 < AE < 1.05, including the target genes and endogenous controls.

To confirm the differential expressions of selected DE miRNAs, we first calculated the fold change between low- and high-altitude goat populations using both relative expression and TMM-normalized read counts. Next, we calculated the Pearson’s correlations between fold change of relative expression and TMM-normalized read counts. miRNAs with a Pearson’s correlation <0.05 were confirmed as differential expressed.

### Luciferase Reporter Assay

Based on identified high-confidence target pairs, we selected 9 DE miRNA–mRNA pairs, including miR-409-5p/*PLCG2*, miR-409-5p/*CDK6*, miR-409-5p/*FLT1*, miR-210-3p/*PIK3R3*, miR-106a-5p/*FLT1*, miR-509-3p/*CAMK2G*, miR-2355-5p/*SERPINE1*, miR-208a-3p/*CAMK2G*, and miR-30c-5p/*PHKB* for target validations. Luciferase activity assays were performed to validate the potential relationship between miRNAs and their target genes. In detail, 3’-UTR sequences containing the miRNA binding site of nine candidate target genes were synthesized and then inserted into the multiple cloning site of pmirGLO plasmid. The recombinant pmirGLO vector and miRNA mimic or NC was cotransfected into HeLa cells at 60% confluency using Lipofectamine 3000 (Invitrogen, Grand Island, NY, United States), according to the manufacturer’s instructions. After transfection for 48 h, dual luciferase activity was tested by Luciferase Dual Assay Kit (Promega, Madison, WI, United States). Luciferase activity was expressed as an adjusted value (firefly normalized to renilla).

### Cell Culture and miRNA Transfection

Human umbilical venous endothelial cells were obtained from the cell bank of the Chinese Academy of Sciences and routinely maintained in DMEM (Hyclone, Logan, UT, United States) supplemented with 10% FBS (GIBCO, Grand Island, NY, United States) at 37°C, 5% CO_2_. According to experimental requirements, the specific inhibitor of miR-106a-5p and NC were purchased from RiboBio (Guangzhou, China). Transfection of miRNAs into HUVECs at 70% confluency was performed using Hiperfect (QIAGEN, Germany) in accordance with the manufacturer’s protocol. After 6 h in transfection, all groups were replaced with new medium for subsequent experimentation.

### Scratch and Tube Formation Assay

Human umbilical venous endothelial cells were cultured to near confluence in 12-well plates. Cell monolayer were straight scratched with a 10 μL pipette tip. PBS gently washed three times to remove the non-adherent cells and cell debris in supernatants. Next, miR-106a-5p and NC were transfected into HUVECs. Photos were taken at the same fields of view at 0, 12, and 24 h after scratching using an Olympus IX53 microscope (Olympus, Tokyo, Japan). For tube formation analysis, the transfected HUVECs were collected and added to the matrigel pre-polymerized well in the 24-well plate. The *de novo* formed capillary-like structures were imaged and quantified using ImageJ software (Bethesda, MA, United States).

### Cell Viability and Proliferation

Cell viability and proliferation were assessed using a Cell Counting kit 8 (Beyotime Biotechnology, Guangzhou, China) and Edu staining (RiboBio, Guangzhou, China) analysis according to manufacturer’s protocol, respectively. For CCK8 detection, 10 μL CCK8 reagent was added to the culture medium of the transfected HUVECs. After incubation for 4 h, OD_450_ were measured using a microplate reader (Thermo Fisher Scientific, Madrid, Spain). For Edu analysis, the transfected HUVECs were treated with 10 μM Edu for 24 h and incubated for 14 h. Edu staining was performed according to the manufacturer’s protocol, cell nuclei stained with DAPI. Images were captured using an Olympus IX53 microscope (Olympus, Tokyo, Japan). The ratio of Edu positive cells were calculated from three independent experiments. The data were expressed as mean ± SD.

## Data Availability Statement

The datasets generated for this study can be found in the GEO (GSE125665 and GSE66242).

## Ethics Statement

The animal study was reviewed and approved by Institutional Animal Care and Use Committee in College of Animal Science and Technology, Sichuan Agricultural University.

## Author Contributions

SF, QT, and ML formulated and evolved the overarching research goals and aims. SF, JM, and QT developed and designed the methodology. SF completed all of the data analysis and was a major contributor in writing the manuscript. JM and KL performed the qRT-PCR validation. JZ, WQ, YL, LJ, XW, AJ, LL, and WX provided reagents, materials, laboratory samples, instrumentation and contributed to acquisition of animal information. SF, JM, and KL wrote the original manuscript. JM and ML reviewed and edited the manuscript. QT and ML took oversight and leadership responsibility for the research activity planning and execution. ML acquired the financial support for the project leading to this publication. All authors read and approved the final manuscript.

## Conflict of Interest

The authors declare that the research was conducted in the absence of any commercial or financial relationships that could be construed as a potential conflict of interest.

## Supplementary Material

FIGURE S1 Length distribution of sequenced high quality reads for each sample. Each curve represents a sample.

FIGURE S2 Number of mappable reads of samples from high- and low-altitude goat populations. Each dot represents a sample. Red and green boxes indicate high- and low-altitude samples, respectively.

FIGURE S3 Genomic sources of miRNA precursor sequences. Each proportion of this pie chart and the corresponding color represents a type of genomic elements. ‘Ambiguous’ refers to miRNA precursor sequences that locate in both introns and exons due to alternative splicing of protein-coding genes. The percentage beneath the black line represented the expected proportion based on the fraction of the genome that corresponds to the considered genomic element, and the *P* value was calculated by permutation test.

FIGURE S4 Distribution curve for the frequency of TSI of **(A)** DE miRNAs and **(B)** non-DE miRNAs.

FIGURE S5 Number of DE miRNAs with tissue-specificity and overlap in tissues. The *P* value was calculated using the Pearson’s Chi-squared test with Yates’ continuity correction.

FIGURE S6 Dual luciferase reporter assay performed on nine candidate miRNA-mRNA pairs to validate the potential target relationship.

TABLE S1 RNA quality of all samples. The RNA integrity number (RIN) was used for determining RNA quality.

TABLE S2 Functional enrichment of genes targeted by tissue-specific miRNAs which differentially expressed with no overlap among tissues.

TABLE S3 High-confidence target genes participated in high-altitude-related response according to the literature.

TABLE S4 Expression alteration of miRNAs and target genes involved in pathways related to high-altitude acclimatization.

TABLE S5 qRT-PCR validations of DE miRNAs involved in pathways related to high-altitude acclimatization. The Pearson’s correlations were calculated between fold change (relative expression) and fold change (TMM-normalized read counts). The fold changes were calculated between low- and high-altitude goat populations within each tissue.

TABLE S6 Primer sequences for qRT-PCR.

## Abbreviations

DE: differentially expressed
DEGs: differentially expressed genes
FPKM: fragments per kilobase of exon per million fragments mapped
GEO: Gene Expression Omnibus
GO: Gene Ontology
HUVECs: human umbilical venous endothelial cells
KEGG: Kyoto Encyclopedia of Genes and Genomes
NC: negative control
PCA: principal components analysis
qRT-PCR: quantitative real-time PCR
RIN: RNA integrity number
RPM: reads per million
TMM: trimmed mean of M-values
TSI: tissue specificity index
TSS: tissue specificity score
UV: ultraviolet radiation.
