# Supplementary figures and images for: Comparative microRNA Transcriptomes in Domestic Goats Reveal Acclimatization to High Altitude
Source: Front Genet. 2020 Jul 31;11:809. doi: 10.3389/fgene.2020.00809 (PMC7411263; doi:10.3389/fgene.2020.00809)

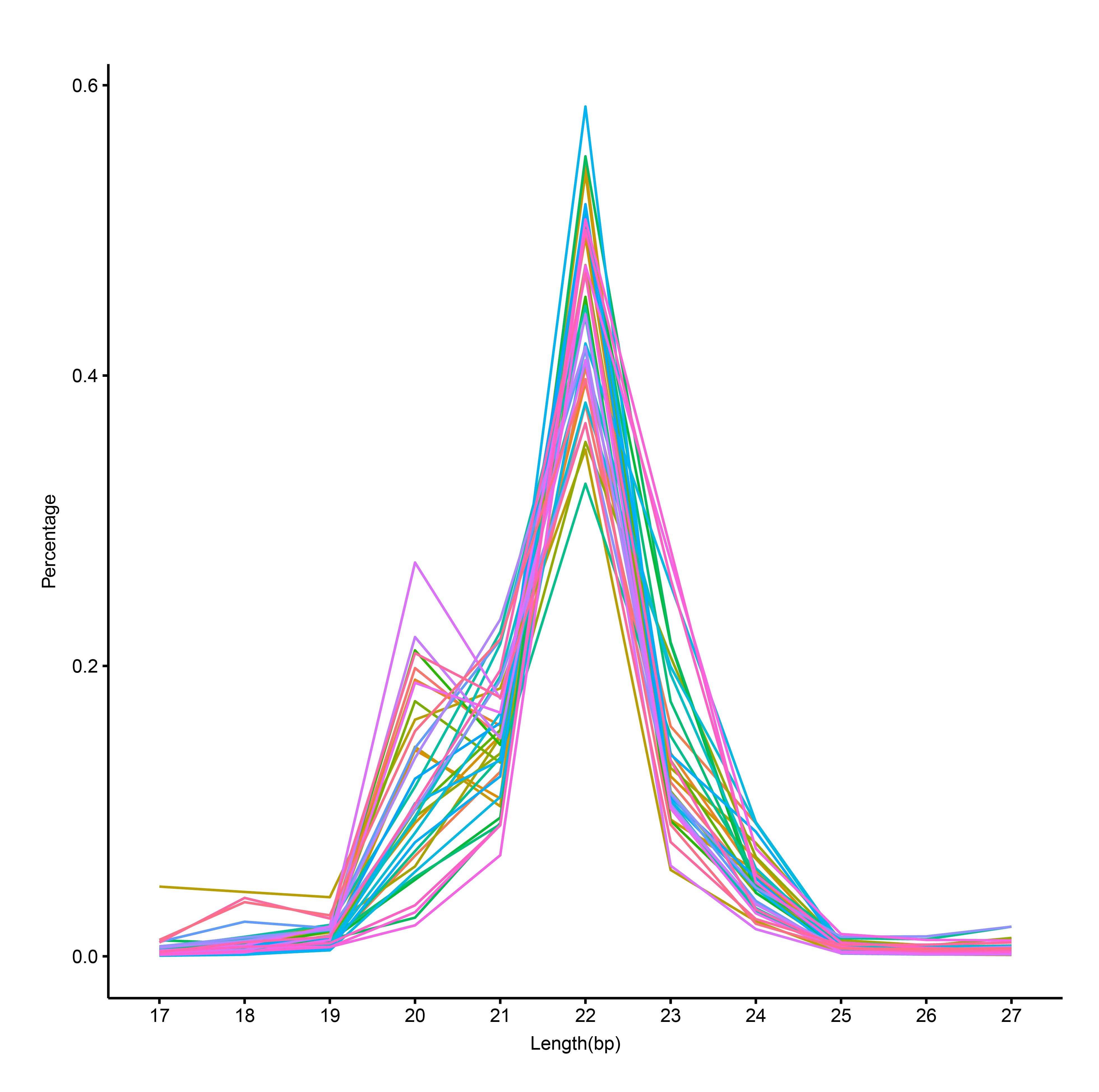

Supplement: FIGURE S1 — Length distribution of sequenced high quality reads for each sample. Each curve represents a sample. [file Image_1.JPEG]

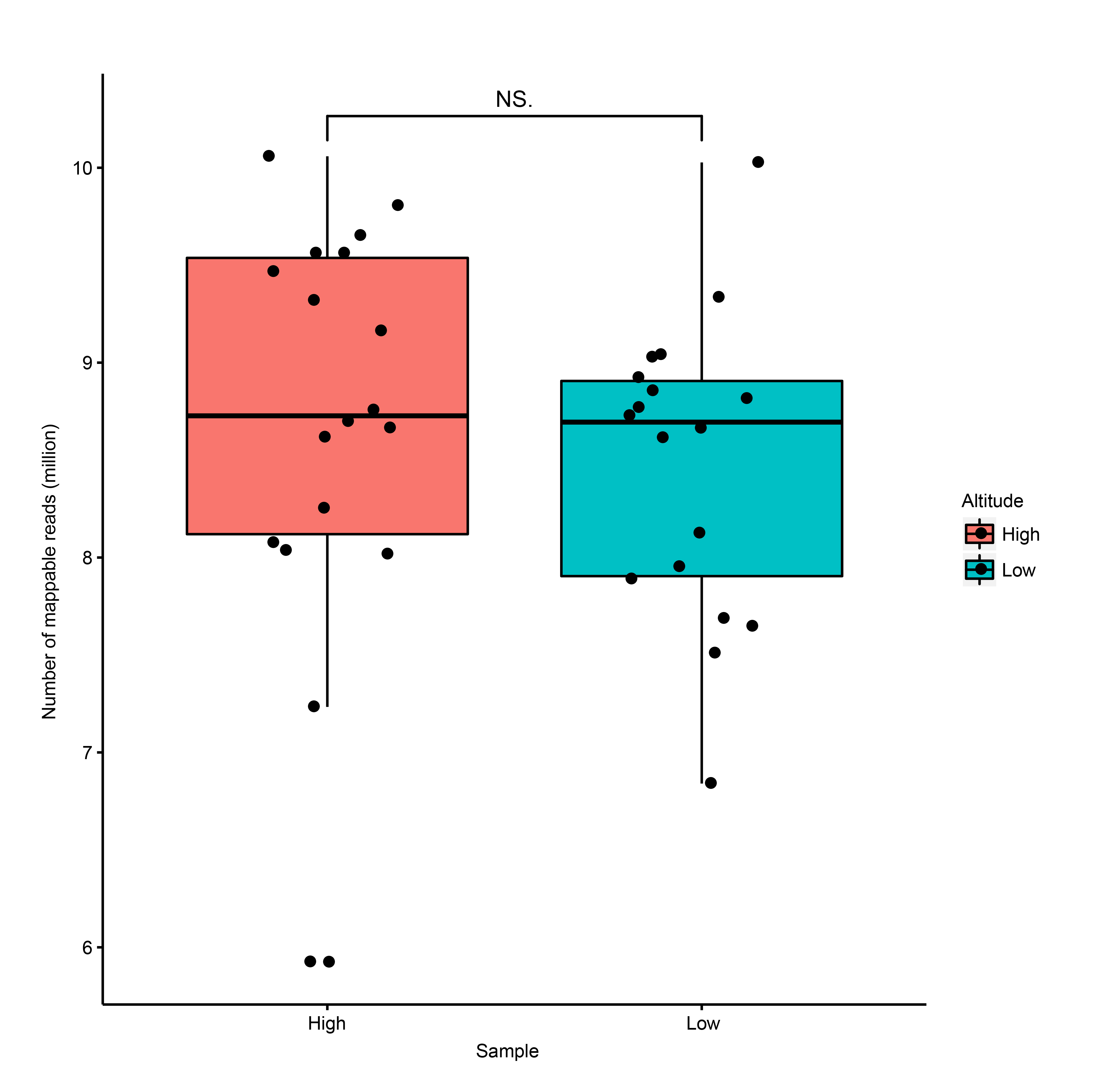

Supplement: FIGURE S2 — Number of mappable reads of samples from high- and low-altitude goat populations. Each dot represents a sample. Red and green boxes indicate high- and low-altitude samples, respectively. [file Image_2.JPEG]

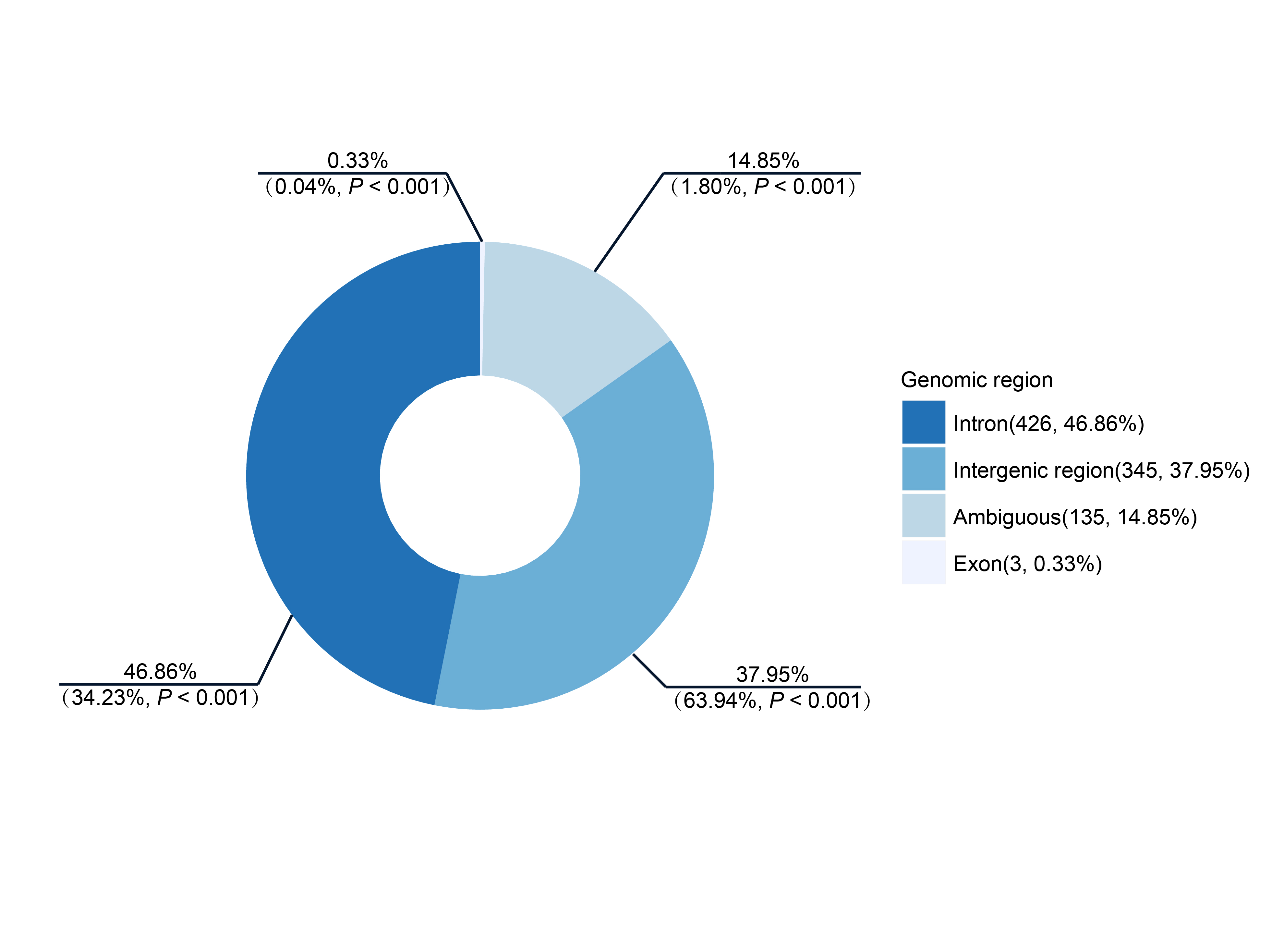

Supplement: FIGURE S3 — Genomic sources of miRNA precursor sequences. Each proportion of this pie chart and the corresponding color represents a type of genomic elements. ‘Ambiguous’ refers to miRNA precursor sequences that locate in both introns and exons due to alternative splicing of protein-coding genes. The percentage beneath the black line represented the expected proportion based on the fraction of the genome that corresponds to the considered genomic element, and the P value was calculated by permutation test. [file Image_3.JPEG]

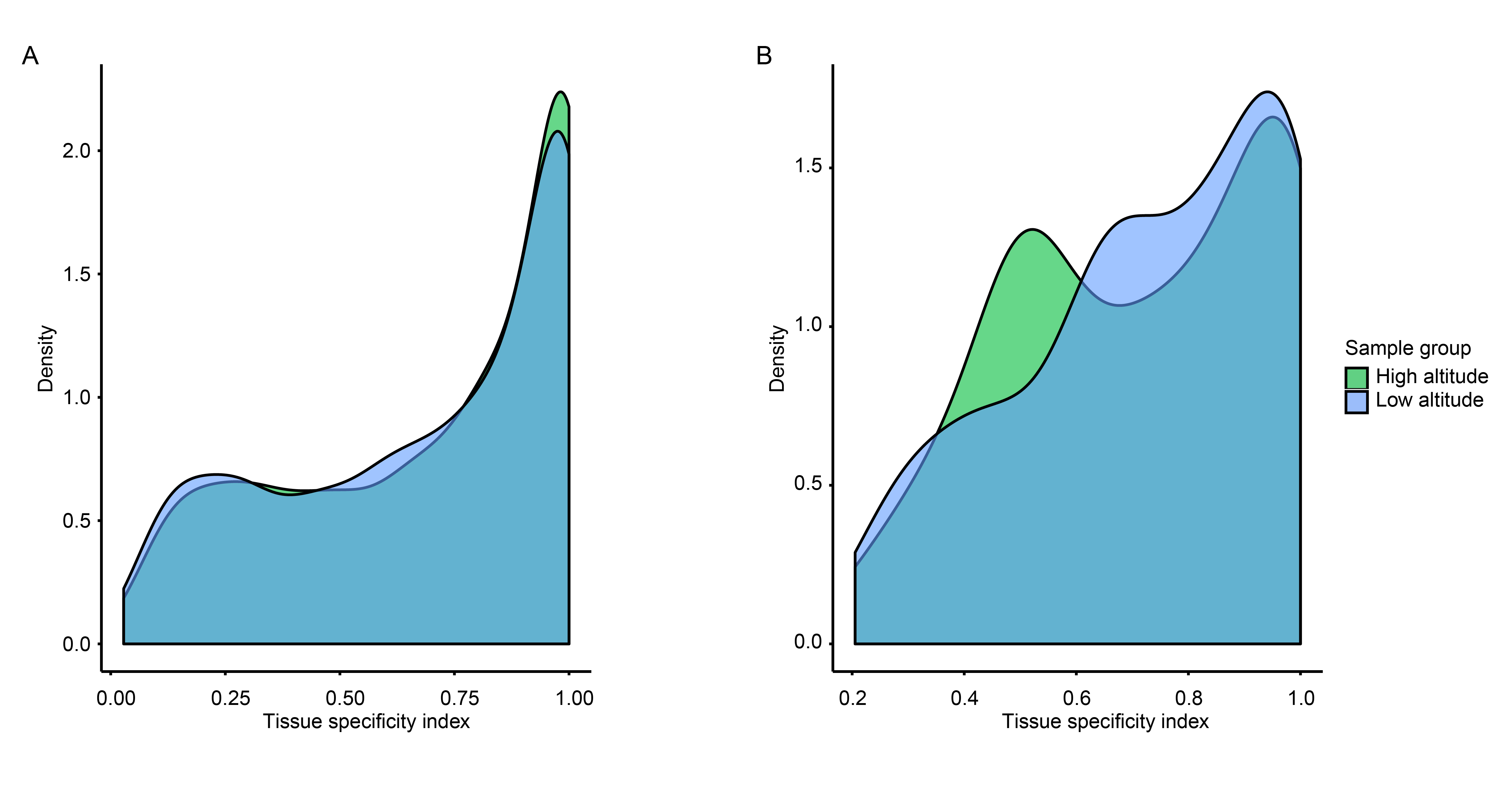

Supplement: FIGURE S4 — Distribution curve for the frequency of TSI of (A) DE miRNAs and (B) non-DE miRNAs. [file Image_4.JPEG]

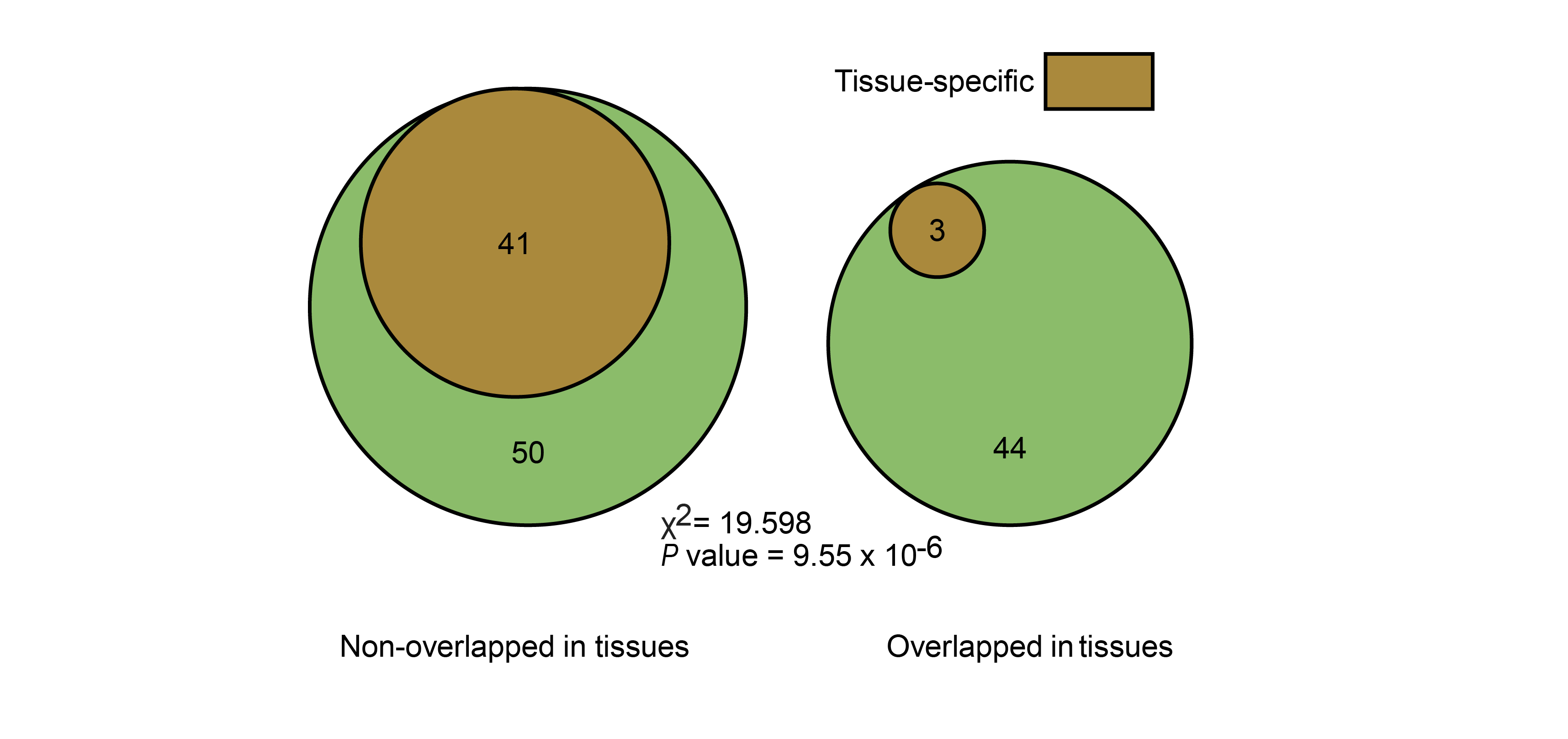

Supplement: FIGURE S5 — Number of DE miRNAs with tissue-specificity and overlap in tissues. The P value was calculated using the Pearson’s Chi-squared test with Yates’ continuity correction. [file Image_5.JPEG]

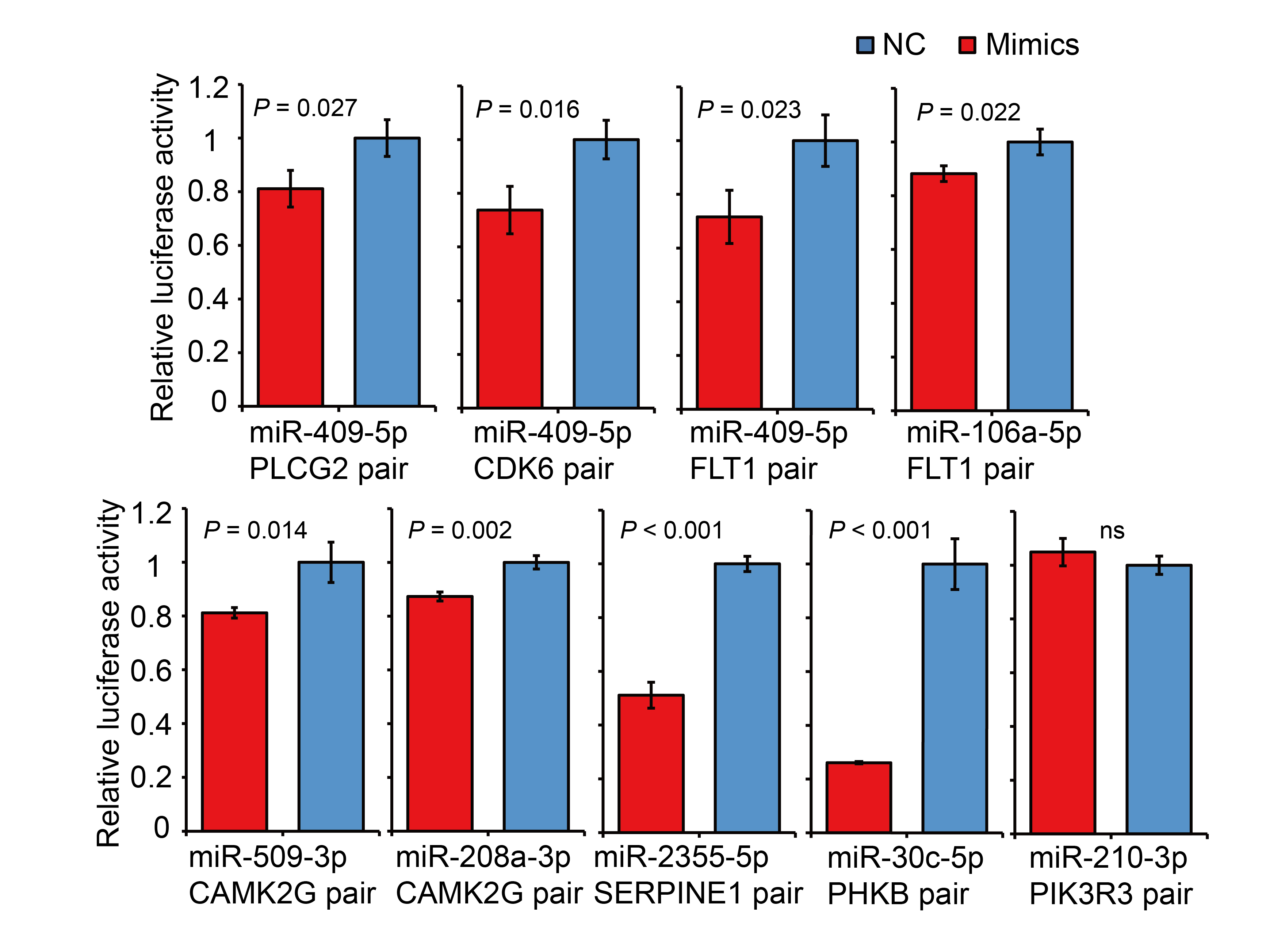

Supplement: FIGURE S6 — Dual luciferase reporter assay performed on nine candidate miRNA-mRNA pairs to validate the potential target relationship. [file Image_6.JPEG]
